# An Augmented Classical Least Squares Method for Quantitative Raman Spectral Analysis against Component Information Loss

**DOI:** 10.1155/2013/306937

**Published:** 2013-07-08

**Authors:** Yan Zhou, Hui Cao

**Affiliations:** ^1^School of Energy & Power Engineering, Xi'an Jiaotong University, Xi'an 710049, China; ^2^State Key Laboratory of Electrical Insulation and Power Equipment, School of Electrical Engineering, Xi'an Jiaotong University, Xi'an 710049, China

## Abstract

We propose an augmented classical least squares (ACLS) calibration method for quantitative Raman spectral analysis against component information loss. The Raman spectral signals with low analyte concentration correlations were selected and used as the substitutes for unknown quantitative component information during the CLS calibration procedure. The number of selected signals was determined by using the leave-one-out root-mean-square error of cross-validation (RMSECV) curve. An ACLS model was built based on the augmented concentration matrix and the reference spectral signal matrix. The proposed method was compared with partial least squares (PLS) and principal component regression (PCR) using one example: a data set recorded from an experiment of analyte concentration determination using Raman spectroscopy. A 2-fold cross-validation with Venetian blinds strategy was exploited to evaluate the predictive power of the proposed method. The one-way variance analysis (ANOVA) was used to access the predictive power difference between the proposed method and existing methods. Results indicated that the proposed method is effective at increasing the robust predictive power of traditional CLS model against component information loss and its predictive power is comparable to that of PLS or PCR.

## 1. Introduction

When lasers were improved as well as the corresponding instrumental techniques, a stable and useful Raman spectrum could be obtained, and thus, it has become a feasible technique for online analysis of reacting systems [1]. One important application of Raman spectroscopy is to measure the analyte concentration, and such studies using liquid-core optical fibres have been investigated [2, 3]. As an analytical tool, the Raman spectroscopy is always combined with suitable chemometric calibration methods to achieve accurate predictions. Recent decades have seen the rapid development in the area of chemometrics for quantitative spectral analysis [4]. In general, two main approaches were focused on: the intuitive calibration method [5, 6] and the inverse/implicit calibration method [7, 8], which were both used to extract informative signals from the spectra and build the relationship between spectral signals and the corresponding analyte concentrations. These methods are efficient in the analyte quantitative analysis because of avoiding a prior separation due to the signal overlapping [6]. 

The intuitive method is based on the Beer-Lambert Law, in which each mixed spectral signal is deemed as a linear combination of all pure component spectral signals at the same wavelength [5]. The most typical intuitive method is classical least squares (CLS), which needs all the component information contributing to the spectral signal [6]. The component information not only includes analyte concentrations but also contains system nonlinearity [9], temperature effects [10], and wavelength shift [11]. In practice, not all such component information can be obtained. In particular, when using Raman spectroscopy for quantitative analysis, the system nonlinearity and wavelength shift are often inevitable, and it is hard to know their levels. With component information loss, CLS model may yield poor predictions. More precisely, if only one component is not considered for the CLS calibration, then the CLS model will not provide accurate predictions for all the analytes [12]. The level of inaccuracy for the CLS model predictions depends on the amount of spectral variance introduced by the unconsidered components during the calibration procedure [13]. For the reasons mentioned above, the applications of CLS were limited and received little further attention recently. 

In contrast, inverse/implicit methods do not have such deficiencies. The main inverse/implicit methods for spectral signal calibration are partial least squares (PLS) [7] and principal component regression (PCR) [8]. In these methods, the analyte concentrations to be determined are regarded as the linear combinations of obtained spectral signals. The core idea of these algorithms is to reduce the dimensionality of a data set in which there are a large number of interrelated variables, while retaining as much as possible of the variation present in the data set. This reduction is achieved by transforming the data to produce a new set of variables, which are uncorrelated, and these components contain the most variance information in the data set [14]. In this context, the inverse/implicit methods can estimate parametric relationships between these variables without requiring a matrix inversion, which is typically the case for CLS. Inverse/implicit methods can offer accurate prediction results in the situation where only the component(s) of interesting is considered. Therefore, they were much more popular in different applications [15–17] recently. 

The CLS calibration method has been analyzed to find out the factor which renders poor predictions for the calibration model [18–20]. It has been proved that the pure spectral signal vectors corresponding to the unmodeled components decrease the predictive power of CLS calibration model [20]. If these vectors are not orthogonal to the pure spectral vectors of the components of interesting, then they may distort the original subspace for CLS modeling and provide inaccurate predictions. To augment the predictive power of the traditional CLS model, several approaches have been developed. A literature review shows that two ways can be distinguished: the first one tries to augment the concentration matrix during the CLS calibration procedure [20], while the second one aims to augment the pure component spectral signal matrix [21–24]. Both approaches need to use the prior knowledge or the supplies from inverse/implicit methods like PLS or PCA to avoid the risk mentioned above. It was reported that the predictive power of these methods is comparable to that of PLS or PCR. It should be noted that the component quantitative information also contains system nonlinearity or wavelength shift, and the pure spectra of such components are hard or impossible to be obtained. For this reason, the augmented classical least squares (ACLS) methods based on pure component spectra cannot be used in many cases. The alternative ACLS methods using inverse/implicit methods can also produce accurate predictions, but they are even more complicated than the original inverse/implicit methods, which may decrease the computation speed for the algorithms. Existing ACLS methods show considerable promise for improving multivariate spectral calibrations, but they still require the supply from prior knowledge or inverse/implicit methods.

In this paper, we propose a concise ACLS method without using the pure component spectra or inverse/implicit methods to overcome the nonlinearity from the Raman spectroscopy. The spectral signals with low analyte concentration correlations were directly put into the concentration matrix to compensate the information loss from unknown components. One data set was used to assess the method. The predictive power of the proposed method was analyzed and compared with PLS and PCR based on a one-way variance analysis (ANOVA) [25].

## 2. Method and Data Set

### 2.1. Augmented Classical Least Squares (ACLS)

In this study, a spectrum corresponding to a mixture of *N* + 1 analytes was defined as a vector **a**  (**a** ∈ *R* ^1×*P*^), and each element in **a** represents a measured spectral signal intensity. **a** can be expressed as

(1) $$\begin{array}{c} a=cK+\varepsilon , \end{array}$$

where each element of **c** refers to a component quantitative value (concentration or other quantitative value for a component) contributing to the signal variance of **a**. **K**  (**K** ∈ *R* ^(*N*+1)×*P*^) is a matrix of the measured spectral intensities (variable), and each row of **K** represents a vector of spectral intensities corresponding to the referred element of **c**. **ε**  (**ε** ∈ *R* ^1×*P*^) is the noise vector. When *L* observations are obtained, (1) can be expressed as

(2) $$\begin{array}{c} A=CK+E, \end{array}$$

where **A**  (**A** ∈ *R* ^*L*×*P*^) is the obtained spectral signal matrix, and each row in **A** refers to the **a** in (1). **C**  (**C** ∈ **R** ^*L*×(*N*+1)^) is the obtained component quantitative matrix, and each row in **C** refers to the **c** in (1). The **K** in (2) is the same as the **K** in (1). **E**  (**E** ∈ *R* ^*L*×*P*^) is the noise matrix, and each row in **E** refers to the **ε** in (1). Thus, the fitting value of **K** can be obtained using

(3) $$\begin{array}{c} \hat{K}={\left(C^{T}C\right)}^{-1}C^{T}A. \end{array}$$

In (3), quantitative values of all the components should be considered, otherwise the pure-component spectra $\hat{K}$ cannot be accurately predicted (the elements corresponding to the components of interesting in (**C** ^*T*^ **C**)^−1^ will be changed), and the degree of inaccuracy depends on the variance introduced by the unconsidered components [13]. Not all the component information can be obtained in many cases. Therefore, if component loss is present, the row of $\hat{K}$ corresponding to the analyte concentration of interesting will be corrupted, which leads to an inaccurate prediction of the analyte concentration of interesting. In this study, spectral signals with low analyte concentration correlations were added into the **C** matrix in (3) as additional columns. The row corresponding to the analyte concentration of interesting may be corrected. Thus, a corrected prediction of the analyte concentration can be made using

(4) $$\begin{array}{c} \tilde{C}=A{\tilde{K}}^{T}{\left(\tilde{K}{\tilde{K}}^{T}\right)}^{-1}. \end{array}$$

In (4), $\tilde{C}$ and $\tilde{K}$ are the corrected $\hat{C}$ and $\hat{K}$, respectively.

### 2.2. Data Set

The Raman data studied in this work were obtained from the catalytic hydrogenation of phenylacetylene (PA) to styrene (ST) and then to ethylbenzene (EB) in a heptane solvent using 5% Pd/CaCO3. The concentrations for the three compounds in each sample were also confirmed using a gas chromatography (GC). The Raman spectra were taken using an Avalon Instruments Raman Station R3, coupled with a fiber optic probe encased in a high pressure sheath. Each Raman spectrum includes more than 1400 signals. The spectral wave number range is 230–3500 cm^−1^, and the minimal spectral resolution is 2 cm^−1^. Preprocessing of the data was performed to remove the background solvent spectral signals. Since the reaction rate is decreasing during the process, the sample concentration values show an uneven distribution. The ranges of the three analytes are 0–0.4598 mol/cc, 0–0.4083 mol/cc, and 0–0.3818 mol/cc, for PA, ST, and EB, respectively. The concentration of PA decreases during the whole process, while the concentration of EB increases during the whole process. The concentration of ST increases to a maximum peak and then decreases. 110 samples in total were obtained from 7 independent processes. In this study, the concentration of PA is the concentration of interesting. Samples were divided into a calibration set and a validation set based on a 2-fold cross-validation with Venetian blinds strategy, in which the similar concentration ranges and correlations for all known components in both the calibration and validation set were present.

### 2.3. Data Analysis

The Raman data set was applied to evaluate the performance of the proposed method. The root-mean-square error of cross-validation (RMSECV) [24] was used to determine the number of added vectors in the concentration matrix. And the root-mean-square error of prediction (RMSEP) [24] was used as the performance criterion to assess and compare the predictive ability of the resulting models. The significance of differences in model performance was given using a one-way analysis of variance (ANOVA) test, which is performed based on the absolute values of the prediction errors. All spectra and concentrations are centered around the mean of the calibration set prior to the analysis. 

## 3. Results and Discussions

**Figure 1:**
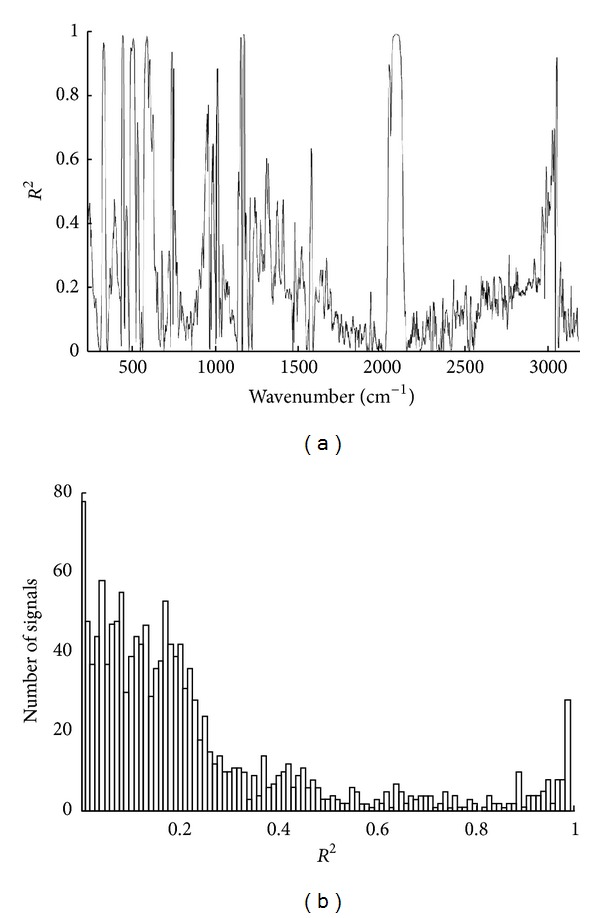
(a) *R* ^2^ values for all signals. (b) Histogram for the *R* ^2^ distribution.

Figure 1(a) shows the squared correlation coefficient (*R* ^2^) values (between the signal and the PA concentration) for all spectral signals, and Figure 1(b) shows the corresponding histogram for the *R* ^2^ distribution. In Figure 1, it is very clear that most of the signals with low *R* ^2^ values (<0.2) are located in the region between 1000 cm^−1^ and 3000 cm^−1^.

Since the samples were obtained from the practical process mentioned above, the concentrations of the three species are not independent mutually. In the calibration set, the *R* ^2^ between the concentrations of PA and ST is 0.24130, and the *R* ^2^ between the concentrations of PA and EB is 0.44169. As can be seen from Figure 1(b), the number of signals corresponding to *R* ^2^ around 0.24130 and 0.44169 is small. It reveals that the signals with high correlation with the two analytes are very few. Thus, it is difficult to find the signals having high *R* ^2^ values for ST and EB without any prior knowledge. It can also be seen that the number of signals with *R* ^2^ ranging from 0.2 to 0.8 is not very large, which suggests that the signals evenly depending on all the three species are limited in number. The *R* ^2^ values of most signals are lower than 0.2, and such signals distribute around the entire spectral signal region. These results indicate that most of the low *R* ^2^ signals contain unknown component variance information. In particular, many signals are with *R* ^2^ around 0, which suggests independent components are present in the samples. As analyzed above, it is impractical to find signals with good ST/EB correlations, so in this study, we try to find the signals (to be added into **C**) in the signal group with low *R* ^2^ values. Since the signals with low *R* ^2^ have poor correlations with PA, it is very possible that such signals are the linear combinations of the concentrations of ST and EB. Consequently, adding the signals into **C** of (3) can also be effective at augmenting the predictive power of CLS model. It should be noted that the signals with low *R* ^2^ values may have good relationship with other unconsidered components (excluding the concentrations of ST and EB), and therefore such signals can be used to substitute the original unconsidered component quantitative information for CLS modeling. It is also possible that the signals with very low *R* ^2^ will introduce orthogonal noises. However, it has been analyzed in [20] that the signal vectors orthogonal to the analyte concentration vector cannot decrease the prediction power of CLS model. Thus, the selection of such signals will not decrease the accuracy of prediction. Since the signals with low *R* ^2^ are closed to each other in the spectrum, they may have high correlationships mutually, which may render redundant signal selections. In this work, to avoid redundant selections, signals were selected from different wave number regions.

**Figure 2:**
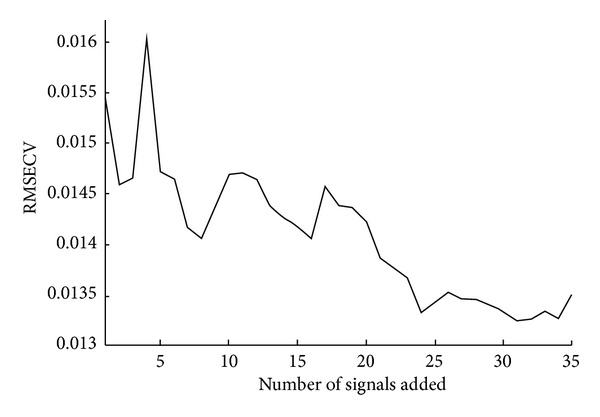
RMSECV versus the number of added signals.

Figure 2 depicts the value of leave-one-out root-mean-square error of cross-validation (RMSECV) [24] for the CLS model versus the corresponding number of signals added into the concentration matrix **C** in (3). The CLS model shows a very unstable prediction power with the increasing number of added signals. The RMSECV value reaches the maximum as 4 signals were added. It may be explained by the fact that unorthogonal noises were introduced via the added signals. After reaching the maximum of 0.016, the RMSECV value decreases rapidly as the number of added signals increases.

Two local maximum can also be found at 11 and 17, which may be explained as above. In the vicinity at 11, a flat region can be seen. It may indicate that redundant signals were added into the concentration matrix **C** in (3). These redundant signals provide little information for the calibration if the vectors corresponding to them can be the combinations of that existing concentration vectors. Although no minimum RMSECV value can be found in Figure 2, a clear RMSECV curve elbow can be seen at approximately 24 which indicates a suitable number of signals to be added. 

Table 1 shows a prediction power comparison of different methods. Various PA prediction models were built using the five main modeling methods and the proposed method. It should be noted that only CLS2 uses all available compound concentrations for modeling. The root-mean-square error of prediction (RMSEP) and *R* ^2^ for the validation set were used to evaluate the prediction power of each model. RMSECV for calibration set was used to compare the model behaviors. To evaluate the difference level, ANOVA test was carried out using the absolute values of the prediction errors. The *P* values for the one-way ANOVA were provided. 

The proposed method achieved the most accurate prediction, while the traditional CLS model (CLS1 in Table 1) produced the most inaccurate prediction. It is very clear that the predictive power of the proposed method performs much better than the CLS2, and this indicates that besides the concentration information for all analytes known, there are other active unknown components. The best result is achieved with a RMSEP value of 0.01128, which can be confirmed by the corresponding RMSECV value of 0.01303 and *R* ^2^ value of 0.99534. The CLS1 model shown in Table 1, using only the PA concentration, produced the largest RMSEP value of 0.24032. It can be seen that the traditional CLS model is significantly different (*P* < 0.0001) from the CRACLS model, and it confirms that the introduced concentration residuals provide useful information to the model as analyzed in [20]. The result also suggests that the variance information loss from other components is very large; therefore, the lacking of it will definitely result in a poor prediction. 

By checking the RMSEP values of CRACLS and CLS2, it can be found that the predictive power of CRACLS is not significantly from that of CLS2 (*P* < 0.5483). However, the RMSEP value for CRACLS is slightly smaller than that for CLS2. It illustrates that the information from concentration residuals is not enough for augmentation in this case; therefore, it reveals that the added concentration residual vectors cannot be the exact combinations of the unconsidered component quantitative vectors. Consequently, this method cannot correct CLS1 model completely. In this study, the numbers of principal components or latent variables for inverse models (PCR, PLS) are selected based on the method proposed in [26]. The PCR model offers a RMSEP value of 0.01212, which is better than CLS2. Although the difference between PCR and CLS2 is not significant (*P* < 0.1149), it is larger than the difference (*P* < 0.5483) between CRACLS and CLS2. Since PCR can extract most of the variance information from the spectral signals, the information loss from unknown components can be compensated automatically even in the situation where not all the analyte concentrations are known.

The PLS model achieves a RMSEP value of 0.01165, a RMSECV value of 0.01318, and a *R* ^2^ value of 0.99467. And the prediction is not significantly different from the prediction produced using PCR (*P* < 0.3467). Although the proposed method shows the most powerful prediction ability, it does not significantly outperform PLS method (*P* < 0.2568). A comparison between it and CLS2 also reconfirms that other components (besides ST and EB) also contribute to the spectral signal variance. The proposed method successfully extracts the lost information from the signals with low *R* ^2^ values.

## 4. Conclusions

The proposed method is able to augment the prediction power of traditional CLS model for quantitative Raman spectral signal analysis. The spectral signals with low analyte concentration *R* ^2^ values can be used to compensate the information loss from unconsidered components. The number of added signals can be determined using the RMSECV curve. The added signals may introduce noises or redundant information to the concentration matrix for CLS modeling. With enough selected signals, the prediction can be improved dramatically. The prediction of the proposed method is more accurate than the traditional CLS method using all analyte concentrations, and it is comparable to the prediction obtained using PLS or PCR.

## Figures and Tables

**Table 1:** Prediction power comparison.

Method	Validation set			Calibration set
	*R* ^2^	RMSEP	*P* value	RMSECV
*CLS1 *	*0.02818 *	*0.24022 *	—	*0.244141 *
CRACLS	0.99423	0.01275	<0.0001	0.01573
CLS2	0.99429	0.01232	0.5483	0.01564
PCR	0.99417	0.01212	0.1149	0.01412
PLS	0.99467	0.01165	0.3467	0.01318
Proposed method	0.99534	0.01128	0.2568	0.01303

The models are sorted according to increasing prediction power, and the *P* values for the significance testing by a one-way ANOVA of the improvement compared to the previous model are given.

CLS1: classical least squares using only the analyte concentration of interesting.

CLS2: classical least squares using all analyte concentrations.

PCR: principal component regression.

PLS: partial least squares.

CRACLS: concentration residual augmented classical least squares.

RMSECV: root-mean-square error of cross-validation.

RMSEP: root-mean-square error of prediction.
